# Clinical Laboratory Networks Contribute to Strengthening Disease Surveillance: The RESAOLAB Project in West Africa

**DOI:** 10.3402/ehtj.v6i0.19960

**Published:** 2013-01-25

**Authors:** Josette Najjar-Pellet, Jean-Louis Machuron, Flabou Bougoudogo, Jean Sakandé, Iyane Sow, Christophe Paquet, Christophe Longuet

**Affiliations:** 1 Fondation Mérieux, France; 2 Ministry of Health, Mali; 3 Ministry of Health, Burkina Faso; 4 Ministry of Health, Senegal; 5 Agence Française de Développement, France

Sufficient laboratory capacity is essential to effective infectious disease surveillance and control. This is recognized in the current International Health Regulations (IHR), which identify laboratory services as a category of core capacities that all the World Health Organization (WHO) Member States are expected to develop and maintain (1). IHR Core Capacity 8 requires laboratory services for every phase of real-time event management (i.e., detection, investigation, and response), with sample analysis being performed either domestically or through collaboration centers (2).

Laboratory services are considered a key component of national health systems, with the Integrated Disease Surveillance and Response (IDSR) utilizing the structures, processes and personnel of national clinical laboratory services for disease surveillance. However, laboratory services for both patient care and disease surveillance remain among the most neglected components of the overall health system in resource-poor countries. Challenges include lack of national laboratory policy and strategic planning, insufficient numbers of trained professionals, poor laboratory infrastructures, and absence of quality management systems (3).

Thus, several calls have been put forth to improve laboratory capacity in resource-poor countries. In 2008, representatives of African governments, local and international partners participated in a consensus meeting on clinical laboratory in Maputo, Mozambique. Meeting participants called on national governments to develop national laboratory policies and to provide laboratory support for diseases of public health importance; and they called on donors and development partners to commit to work collaboratively with each other and with coordination from national governments to strengthen laboratory systems (4). The WHO Regional Office for Africa (WHO/AFRO) has also advocated strengthening national public health laboratories (5). Also in 2008, WHO and the US Centers for Disease Control and Prevention (CDC), Atlanta, USA, convened in Lyon, France, an international conference on laboratory quality systems. During that meeting, the need for accurate laboratory testing was stressed, with poor quality laboratory services in resource-constrained countries leading to untold misery in human lives and unnecessary expenditures due to inadequate treatment (6). Eight key interventions were identified: (i) strengthening laboratory management at all levels; (ii) strengthening infrastructure and support systems; (iii) developing human capacity; (iv) establishing a national laboratory referral network; (v) establishing a national quality assurance program; (vi) developing a comprehensive monitoring system including laboratory information management system; (vii) coordinating government and partner support activities; and (vii) mobilizing resources to finance the strategic plan. The need to integrate networks that already exist – mostly those related to malaria, tuberculosis and HIV/AIDS – was also stressed.

In response to these calls, several international development partners have been implementing capacity building programs that include the training of laboratory personnel in epidemiology (7), microbiology (8) and quality assurance (9). In 2005, Fondation Mérieux, with the support of the European Commission, launched a national laboratory network initiative in Mali called Action BIOMALI. In just four years, the network grew to cover more than eighty public and private laboratories. In 2009, in response to official demands from the Ministries of Health of two neighboring countries, Burkina Faso and Senegal, and with the support of the French Development Agency (AFD) and Fondation Mérieux, the Mali network was expanded into a three-country regional network called RESAOLAB. This article describes the major activities and accomplishments of RESAOLAB and presents RESAOLAB as an example of how disease surveillance capacity can be built using a regional network strategy.

Established in 1967, Fondation Mérieux is an independent family foundation. Recognized as a state-approved charity in Lyon, France, the Foundation works to reduce the impact of infectious diseases that affect developing countries and currently operates in four countries. Fondation Mérieux prioritizes partnerships and catalyzes both local and international initiatives aimed at helping researchers and health care workers in developing countries learn how to use the best available scientific and medical tools so that they can meet their countries’ public health needs in the long term and independently. Based on its long history of expertise in clinical biology and a comprehensive approach to public health, the Foundation's work serves as a model for strengthening local laboratory capacities.

## RESAOLAB

RESAOLAB strengthens the quality of clinical laboratory services through inter-country meetings and workshops that promote the exchange of knowledge and experiences and harmonization of documents and tools. The network focuses on three main areas of activity: training laboratory personnel, setting quality assurance, and strengthening epidemiological surveillance.

### Training laboratory personnel

The three network countries jointly developed a shared national strategic plan for continuous education of laboratory technicians; all three Ministries of Health officially validated the plan. The countries also jointly wrote content for the continuous education program, which includes a total of nine training modules. In each country, four reference structures were set up to organize training sessions, with a focus on use of harmonized equipment. To date, a total of 64 training sessions have been conducted, with 25 participants per session. The training modules are available for self-training through the GLOBE portal (10).

### Setting quality assurance

As with the laboratory technician training, the three network countries jointly developed a shared national plan for laboratory quality management, which was then validated and adopted by all three Ministries of Health. The document defines standards for personnel organization, laboratory equipment, procedures, data processing, and hygiene and security. Additionally, the network identified and equipped four laboratories in each country responsible for maintaining external quality control. To date, the network has conducted more than 350 supervised external quality control assessments to evaluate the quality of diagnostics being used and identify necessary corrective measures.

### Strengthening the epidemiological surveillance system

Improving the management and the quality of laboratory data has a direct impact on the epidemiological surveillance system. RESAOLAB developed an open-source Laboratory Information and Management System (Lab-Book) for monitoring all daily surveillance activities (from requests for analysis to reporting). Based on jointly defined reference terms, Lab-Book contains an epidemiological application for reporting laboratory data; in collaboration with WHO/AFRO, RESAOLAB conducted a regional workshop to discuss use of the new tool, including the role of the laboratory in reporting epidemiological data to surveillance databases. Fifteen laboratories in each country are expected to participate in a pilot launch of Lab-Book. The network also proposed computer and other equipment, Internet services, and training that will be necessary for integrating Lab-Book across the region.

### Key achievements

In addition to the activities described above, three other key achievements are worth noting here. First, after many meetings advocating for laboratory governance and following Burkina's lead, Senegal established a national laboratory department under the Ministry of Health. Second, during a cholera outbreak in Mali in July 2011, RESAOLAB laboratory technicians played a critical role in the collection and preliminary analysis of surveillance data by directly applied procedures they had learned in the “Epidemic-Prone Diseases in the Laboratory” training module. The outbreak affected nine health districts in Mopti and Timuktu. It started on July 5. By August 4,463 cases had been reported, with a case fatality rate of 5.18 percent (24 deaths). Finally, recognizing the value of regional laboratory networking, four other countries in the region – Benin, Guinea, Togo, and Niger – have made requests to their Ministries of Health to join RESAOLAB.

## Conclusion

RESAOLAB grew from national and regional dialogue around the need for harmonized tools and processes. It serves as a model for groups of neighboring countries that would like to strengthen the laboratory component of their disease surveillance infrastructure by jointly developing and implementing trainings and other activities and by harmonizing and linking national databases into integrated regional systems. As described elsewhere in this special issue of *Emerging Health Threats*, other regions, like South East Europe, are taking similar steps to strengthening disease surveillance laboratory capacity (11).
